# Pioglitazone enhances brain mitochondrial biogenesis and phase II detoxification capacity in neonatal rats with 6-OHDA-induced unilateral striatal lesions

**DOI:** 10.3389/fnins.2023.1186520

**Published:** 2023-07-28

**Authors:** Daniela Vázquez-González, Juan Carlos Corona

**Affiliations:** Laboratory of Neurosciences, Hospital Infantil de México Federico Gómez, Mexico City, Mexico

**Keywords:** ADHD, mitochondrial biogenesis, antioxidant defenses, pioglitazone, 6-OHDA

## Abstract

The psychostimulant methylphenidate (MPH) is the first-line pharmacological treatment for attention-deficit/hyperactivity disorder (ADHD), but has numerous adverse side effects. The PPARγ receptor agonist pioglitazone (PIO) is known to improve mitochondrial bioenergetics and antioxidant capacity, both of which may be deficient in ADHD, suggesting utility as an adjunct therapy. Here, we assessed the effects of PIO on ADHD-like symptoms, mitochondrial biogenesis and antioxidant pathways in multiple brain regions of neonate rats with unilateral striatal lesions induced by 6-hydroxydopamine (6-OHDA) as an experimental ADHD model. Unilateral striatal injection of 6-OHDA reduced ipsilateral dopaminergic innervation by 33% and increased locomotor activity. This locomotor hyperactivity was not altered by PIO treatment for 14 days. However, PIO increased the expression of proteins contributing to mitochondrial biogenesis in the striatum, hippocampus, cerebellum and prefrontal cortex of 6-OHDA-lesioned rats. In addition, PIO treatment enhanced the expression of the phase II transcription factor Nrf2 in the striatum, prefrontal cortex and cerebellum. In contrast, no change in the antioxidant enzyme catalase was observed in any of the brain regions analyzed. Thus, PIO may improve mitochondrial biogenesis and phase 2 detoxification in the ADHD brain. Further studies are required to determine if different dose regimens can exert more comprehensive therapeutic effects against ADHD neuropathology and behavior.

## 1. Introduction

Attention-deficit/hyperactivity disorder (ADHD) is the most common neurodevelopmental disorder in childhood. It is characterized by poor attention, hyperactivity and impulsivity, symptoms, and is typically associated with poor academic, social and psychological function (Corona, 2021; Faraone et al., 2021). The psychostimulant methylphenidate (MPH) is the first-line pharmacological treatment for children with ADHD (Cortese, 2020; Posner et al., 2020). MPH is believed to improve symptoms by blocking presynaptic dopamine and norepinephrine transporters in the brain, thereby enhancing dopaminergic and noradrenergic signaling (Koda et al., 2010; Shellenberg et al., 2020). However, MPH is a controlled substance due to potential for abuse and illegal trafficking and is associated with adverse side effects including nausea, insomnia, anorexia, anxiety, headache, mood instability and dry mouth (Morton and Stockton, 2000; Clemow, 2017; Corona, 2018). These side effects may stem from the induction of oxidative stress and altered antioxidant enzyme activity (Martins et al., 2006; Gray et al., 2007; Gomes et al., 2008), potentially disrupting the activity of brain regions regulating psychological stress, cognition, appetite and motivation. While alterations in dopaminergic neurotransmission are strongly implicated in the pathophysiology of ADHD (Prince, 2008; Del Campo et al., 2011; Gold et al., 2014; Corona, 2018), there is accumulating evidence that mitochondrial dysfunction and oxidative stress also contribute (Verma et al., 2016; Corona, 2020; Ogutlu et al., 2020; Giannoulis et al., 2022). Therefore, improving mitochondrial metabolism and enhancing neural antioxidant capacity may be an effective adjunct therapeutic strategy for ADHD and other neurodevelopmental diseases.

Indeed, mitochondrial dysfunction is implicated in the pathophysiology of multiple neurodegenerative and neuropsychiatric disorders (Corona and Duchen, 2016; Emmerzaal et al., 2021; Rey et al., 2022). Mitochondria generate the ATP required for neuronal excitability, calcium signaling and synaptic plasticity, the essential neural mechanisms underlying cognition and adaptive behavior. However, ATP generation by oxidative phosphorylation also generates reactive oxygen species (ROS) in the brain (Corona and Duchen, 2015a; Nolfi-Donegan et al., 2020). Neurons are rich in mitochondria due to high metabolic demand, and so are prone to ROS accumulation and oxidative damage. Further, mitochondria are highly susceptible to the harmful effects of ROS, leading to oxidative stress, a condition in which endogenous cellular antioxidant capacity is insufficient to prevent uncontrolled ROS accumulation and counteract oxidative damage to essential cellular macromolecules (Forman and Zhang, 2021). In extreme cases, this mitochondrial dysfunction and oxidative stress can lead to cell death (Picca et al., 2020).

Peroxisome proliferator-activated receptor gamma (PPARγ) is a ligand-activated transcription factor of the nuclear hormone receptor superfamily essential for regulation of mitochondrial biogenesis, antioxidant defense, immune responses and glucose metabolism (Corona and Duchen, 2015b, 2016; Jamwal et al., 2021). PPARγ can be activated by glitazone compounds such as pioglitazone, troglitazone and rosiglitazone, all of which are antihyperglycemic drugs used extensively for the treatment of type 2 diabetes. Activation of PPARγ by glitazone agonists increases mitochondrial biogenesis by upregulating the expression of peroxisome proliferator-activated receptor gamma coactivator 1α (PGC1α), mitochondrial transcription factor A (TFAM), nuclear respiratory factors 1 and 2 and various downstream molecules that collectively enhance oxidative phosphorylation capacity (Bogacka et al., 2005; Ghosh et al., 2007; Strum et al., 2007; Miglio et al., 2009). In addition, PPARγ activation upregulates expression of antioxidant enzymes such as catalase (CAT), superoxide dismutase (SOD) and glutathione peroxidase (GPx) (Girnun et al., 2002; Chung et al., 2009; Chen et al., 2012; Corona et al., 2014), as well as nuclear factor erythroid 2-related factor 2 (Nrf2), the master transcriptional regulator of numerous cellular detoxification enzymes (Corona et al., 2014; Lee, 2017). Thus, PPARγ activation may concomitantly enhance protection against oxidative stress from increased mitochondrial metabolism.

Experimental animals with impaired dopaminergic neurotransmission often exhibit phenotypes resembling those of ADHD. For instance, injection of 6-hydroxydopamine (6-OHDA) destroys dopaminergic neurons in the substantia nigra pars compacta (Teicher et al., 1998; Caballero et al., 2011) and leads to ADHD-like behavioral phenotypes such as spontaneous motor hyperactivity, impaired learning, inattention and impulsivity (Zhang et al., 2002; Bouchatta et al., 2018, 2020). Unilateral injection of 6-OHDA into the striatum of neonatal rats was also reported to induce the progressive loss of striatal dopaminergic projections as evidenced by a 35–40% reduction in tyrosine hydroxylase (TH), the rate-limited enzyme in dopamine synthesis, accompanied by a significant decrease in spatial attention, thus validating this rat as an experimental attention deficit-like symptom model (Caballero et al., 2011). Therefore, such animals may be suitable models to examine if adjuvant therapies that counteract bioenergetic deficits or oxidative damage can mitigate ADHD-like behavioral and neurochemical abnormalities. To this end, we tested whether the PPARγ agonist pioglitazone can activate mitochondrial biogenesis and/or antioxidant pathways in various brain regions of neonatal rats lesioned unilaterally by striatal 6-OHDA injection, and thereby reduce ADHD-like behavioral phenotypes.

## 2. Materials and methods

### 2.1. Reagents and antibodies

6-Hydroxydopamine hydrochloride and ascorbic acid were purchased from Sigma-Aldrich (St. Louis, MO) and pioglitazone (Actos TM) was purchased from Takeda Pharmaceuticals (Osaka, Japan). Antibodies targeting PGC1α, TFAM, Nrf2 and catalase as well as ECL substrate were obtained from Santa Cruz Biotechnology (Dallas, TX), Mitobiogenesis Western Blot cocktail, anti-TH and anti-β-actin (1,1000) from Abcam (Cambridge, MA), Entellan from Merck (Darmstadt, Germany) and a DAB substrate kit from BD Biosciences (San Jose, CA). Pioglitazone tablets were crushed into powder and dissolved in 0.9% physiological saline for intragastric administration.

### 2.2. Animals

All experiments were performed using Wistar rats. Pups were housed with their dams under controlled temperature (22°C ± 2°C) and 12 h/12 h light/dark cycle with water and food *ad libitum*. Animal care protocols and experimental procedures were approved by the Hospital Infantil de Mexico Federico Gómez, Institutional Ethical, Animal Care and Use Committees (HIM2018-030). Data are reported in accordance with ARRIVE guidelines. All efforts were made to minimize the number of animals used and their suffering.

### 2.3. Neonatal 6-OHDA lesion on day postnatal 7

On postnatal day 7 (PN7), rat pups were anaesthetized by intraperitoneal (i.p) ketamine-xylazine [50 mg/kg, 5 mg/kg] body weight diluted in 0.9% NaCl, positioned on a stereotaxic frame (Stoelting, Kiel, WI, USA), and injected with 6-OHDA hydrochloride (8 μg/μL calculated from the free base weight in 0.9% NaCl with 0.1% ascorbic acid) into the right dorsolateral striatum (relative to bregma [in mm]: AP = +0.6, ML = −2.5, DV = −3.3) using a 5-μL Hamilton syringe (Hamilton, Reno, NV, USA) at 0.25 μL/min. The syringe needle was left in place for 5 min to allow drug diffusion and avoid any backflow. The control group was injected with vehicle (0.9% NaCl with 0.1% ascorbic acid) using the same procedure. After injection, the pups were warmed to 37°C and returned to their mothers until weaning.

### 2.4. Behavioral testing

Rats were habituated to the testing environments (open field chamber and Y-maze) on postnatal day 22 (PN22) and tested first on day PN25 (before PIO treatment), which is considered the day of prepubertal onset for rats. Behavioral testing was conducted again on PN39 following the indicated drug treatments. Both the open field chamber and Y-maze were cleaned with a 75% ethanol solution before each test to remove any trace of odor.

### 2.5. Open field test

General locomotor activity was assessed in the open field test. Experiments were conducted in an opaque open-topped Plexiglas box (90 × 90 × 90 cm) with a digital camera suspending above. Briefly, individual rats were placed in the center of the apparatus and allowed to move freely for 10 min. Behavior was analyzed using Fiji ImageJ software. Total distance traveled was measured as an indicator of motor activity. All open field tests were conducted between 18:00 and 20:00.

### 2.6. Y-maze test

Spatial working memory performance was examined by recording spontaneous alternation in the Y-maze. The maze was made of opaque Plexiglas and consisted of three connected arms (A, B, C) projecting at 120°, each 45 cm long, 15 cm wide and bounded by walls 15 cm in height. Each rat was placed in the center of the Y-maze (meeting point of the arms) and allowed to explore freely for 10 min. The number of alternations (movements between consecutive arms such as ABC and BCA but not CBC) and the total number of arm entries were recorded and analyzed using Fiji ImageJ software. The % alternation was calculated as the number of alternations / (total number of arm entries −2) × 100. All Y-maze trials were conducted between 17:00 and 19:00.

### 2.7. Animal grouping and pioglitazone treatment

**Figure 1:**
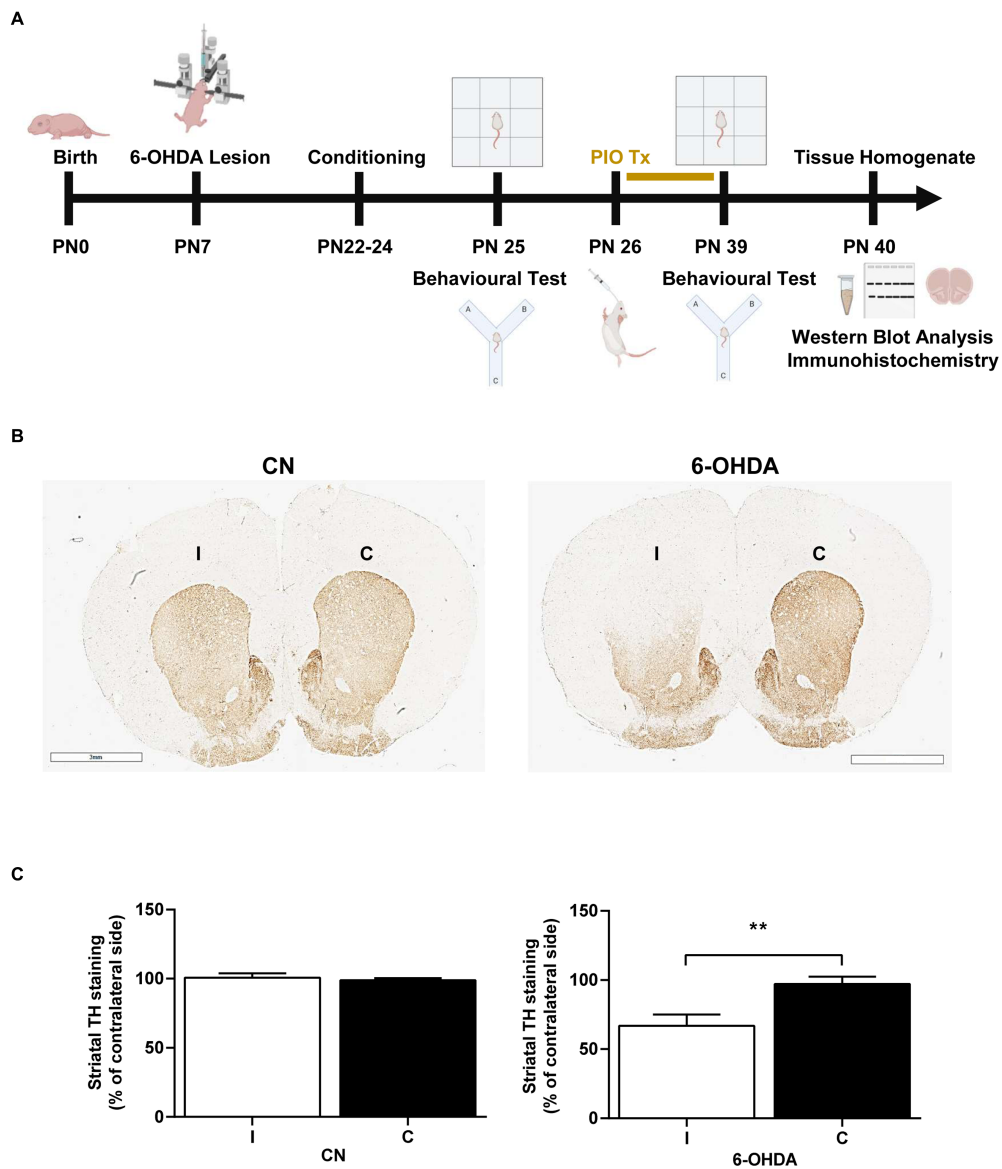
Unilateral injection of 6-OHDA into the rat striatum reduced tyrosine hydroxylase (TH) immunoreactivity on the injection side. **(A)** Schematic representation of experimental procedures and timeline. **(B)** Photomicrographs showing TH immunoreactivity in the ipsilateral (injection side) and contralateral striatum of control rats (CN) injected with vehicle and 6-OHDA-injected rats. In the 6-OHDA group, TH immunoexpression was significantly reduced in the ipsilateral striatum (I) compared to the contralateral striatum **(C)**. Bar = 3 mm. **(C)** Quantification showing a mean 33% decrease in TH immunoexpression compared to the C striatum of 6-OHDA group rats, while immunoexpression was similar between the ipsilateral and contralateral striatum of CN group rats. Results are presented as mean ± SEM of *n* = 8 rats per group. **p < 0.01 vs. contralateral side by Student’s *t*-test.

A total of rats (*n* = 8) were used for the analysis of immunohistochemistry, the other rats were randomly and equally divided into four treatment groups (*n* = 8) on day PN26: (1) A control (CN) group receiving saline (0.9%); (2) a 6-OHDA group; (3) a PIO group receiving 10 mg/kg PIO dissolved in 0.9% saline orally using a feeding needle, once daily for 14 consecutive days (PN26–PN39) (Abdallah, 2010; Ulusoy et al., 2011; El-Sayed et al., 2022); and (4) 6-OHDA + PIO group receiving PIO from PN26–PN39. The control group was treated with saline (0.9%) as a vehicle. Treatment was followed immediately by behavioral retesting (Figure 1A).

### 2.8. Western blot analysis

Hippocampal, striatal and prefrontal cortex tissues from the ipsilateral (four treatment groups) side and contralateral side as well as whole cerebellum were isolated and homogenized separately in RIPA lysis buffer containing an inhibitor cocktail of proteases and phosphatases for 1 h on ice. Lysates were centrifuged at 4°C for 15 min at 12.000 rpm, and supernatants collected. Supernatant protein concentrations were determined using a Bradford reagent. Then, equal amounts of protein were resolved by 10% sodium dodecyl sulfate-polyacrylamide gel electrophoresis and transferred to PVDF membranes (0.22 μM). Membranes were blocked with phosphate buffered saline (PBS) containing 5% non-fat dried milk for 2 h at room temperature (RT), incubated overnight with the indicated primary antibodies in PBS plus Tweet 20 (PBST) at 4°C, rinsed three times in PBST, and incubated with horseradish peroxidase (HRP)-conjugated secondary antibody for 2 h at RT. Immunolabeled bands were detected with chemiluminescence substrate (ECL, Santa Cruz Biotechnology) according to the manufacturer’s instructions using a Fusion-Solo WL imaging system (Vilber Lourmat) and quantified using Fiji ImageJ.

### 2.9. Immunohistochemistry

Immediately after the final behavioral test (PN40), rats were sacrificed by decapitation using a guillotine. Brains were quickly removed, immediately fixed with 4% paraformaldehyde in PBS for 24 h, immersing in 30% sucrose for 5 days, embedded in OCT at −70°C and cut into 20-μm thick coronal sections through the striatum using a cryostat. Free-floating sections were stained for the dopaminergic marker TH. In brief, sections were incubated in 0.3% hydrogen peroxide for 6 min at RT to quench endogenous peroxidase activity, permeabilized by emersion in 0.1% PBST for 10 min (3 times), blocked in PBST supplemented with 1% bovine serum albumin for 2 h and incubated with anti-TH (1:1000) overnight at 4°C. The next day, sections were incubated with a biotinylated secondary antibody for 1 h at RT and stained using a 3-3-diaminobenzidine tetrahydrochloride (DAB) substrate kit (BD Bioscience). Sections were dried, mounted on slides using Entellan and imaged using a ScanScope CS scanner (Aperio Technologies, Vista, CA) and ImageScope software (Aperio).

### 2.10. Statistical analysis

All statistical analyses were conducted using GraphPad Prism Software (Version 8.01, Inc., La Jolla, CA). Data are expressed as the mean ± standard error of the mean (SEM) from at least eight independent experiments. Differences in TH expression between ipsilateral and contralateral sides were compared by Student’s *t*-test. Group differences in behavioral tests were assessed by repeated measures one-way analysis of variance (ANOVA) followed by *post hoc* Bonferroni tests for pair-wise comparisons. Group differences in protein expression were compared by one-way ANOVA with *post hoc* Bonferroni tests. A *p* < 0.01 was considered significant for all tests.

## 3. Results

### 3.1. Unilateral 6-OHDA injection into the striatum on PN7 reduced TH-immunoreactivity

We first evaluated if unilateral injection of 6-OHDA into the striatum of PN7 rats destroyed dopaminergic inputs and depleted striatal dopamine by measuring TH immunoreactivity. Indeed, TH+ labeling in the ipsilateral (I) dorsal striatum was 33% lower than that on the contralateral (C) side by PN39 (Figures 1B,C), while TH+ labeling was similar in both the I and C dorsal striatum of saline-injected control rats (CN group; Figures 1B,C). Therefore, these animals do exhibit a known neurochemical feature of ADHD.

### 3.2. Pioglitazone did not prevent the hyperactivity induced by unilateral 6-OHDA injection into the striatum

**Figure 2:**
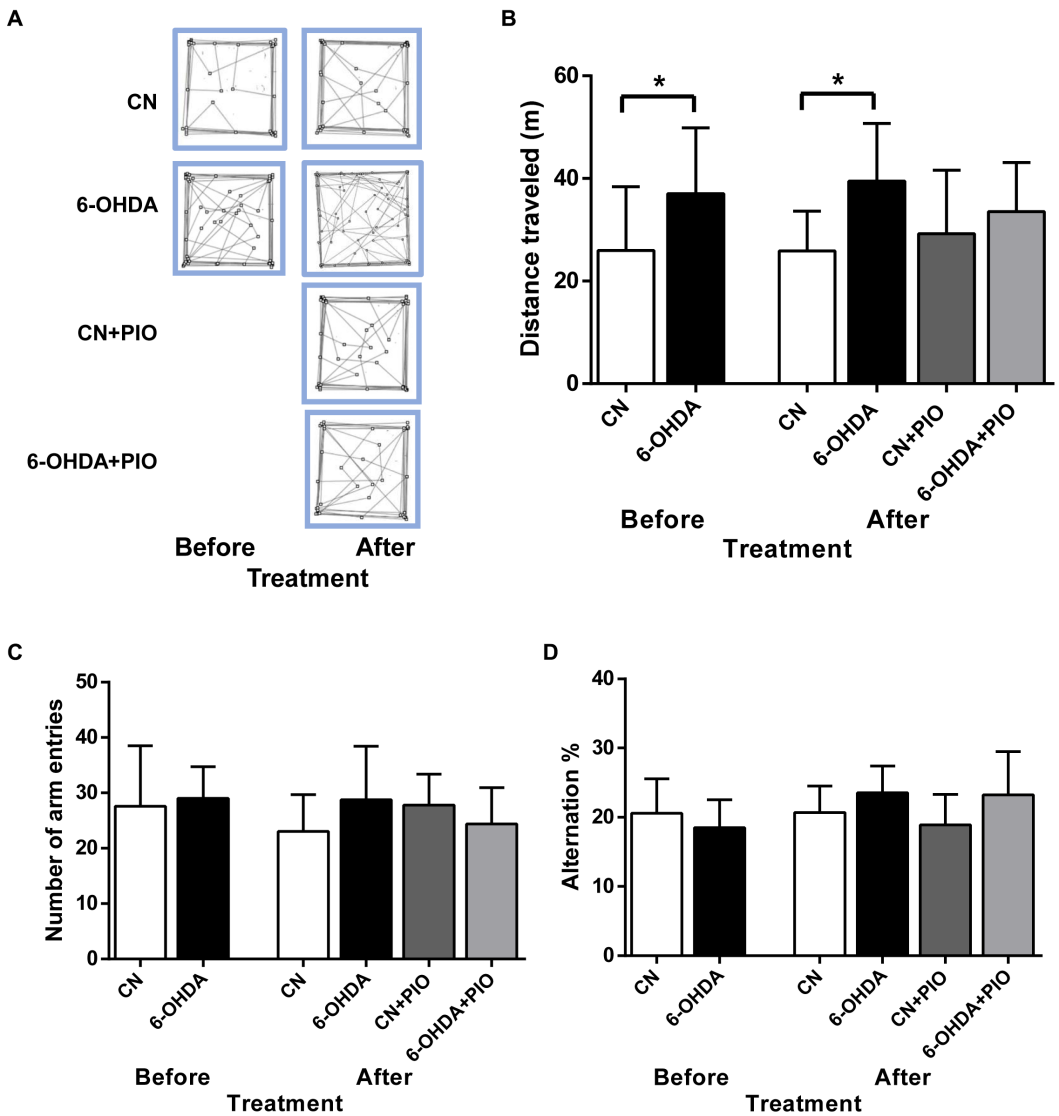
Unilateral 6-OHDA injection induced locomotor hyperactivity that were refractory to pioglitazone (PIO) treatment. **(A)** Representative activity traces of CN and 6-OHDA group rats in the open field (over 10 min) on postnatal day (PN) 25 (before PIO treatment) and PN39 (after treatment with PIO). **(B)** 6-OHDA-lesioned rats exhibited locomotor hyperactivity compared to CN rats both before and after PIO treatment. **(C)** Effects of PIO on the number of arm entries and **(D)** percentage alternation in the Y-maze test. Spatial working memory performance in the Y-maze was not altered by 6-OHDA injection, PIO treatment or both. All results are presented as mean ± SEM of *n* = 8 rats per group. *p < 0.05 vs. CN group by Student’s *t*-test before treatment and by one-way ANOVA with *post hoc* Bonferroni tests after treatment.

We then investigated if these 6-OHDA-injected rats exhibit motor hyperactivity, an ADHD-like behavioral phenotype and the potential ameliorative effects of PIO. On PN25 (before PIO treatment), the 6-OHDA injection group demonstrated significantly greater distance traveled in the open field test compared to control (CN) rats (Figures 2A,B), and this hyperactive phenotype was maintained on PN39 compared to the CN group (Figures 2A,B). However, treatment with 10 mg/kg body weight PIO daily for 14 consecutive days did not induce a statistically significant reduction in distance traveled in the lesioned rats (6-OHDA + PIO group) compared to the 6-OHDA group (Figures 2A,B). Similarly, PIO administration to CN rats (CN + PIO group) had no effect on distance traveled (Figures 2A,B). Thus, this PIO treatment regimen did not prevent locomotor hyperactivity caused by unilateral 6-OHDA injection into the striatum.

### 3.3. Neither unilateral injection 6-OHDA nor pioglitazone influenced spatial working memory

Inattention is one of the major symptoms of ADHD (Corona, 2021), so we evaluated the effects of unilateral 6-OHDA injection into the striatum on spatial working memory using the Y-maze test. However, there was no difference in the number of arm entries between these groups on either test day (Figure 2C). Further, there was no significant difference in % alternation between 6-OHDA and CN groups on either PN25 or PN39 (Figure 2D), indicating no detectable impairment in spatial working memory. Moreover, PIO had no effect on total arm entries or % alternation in either lesioned rats (6-OHDA + PIO group) or CN rats (CN + PIO group) as shown in Figures 2C,D.

### 3.4. Pioglitazone upregulated the expression levels of mitochondrial biogenesis-related proteins following 6-OHDA injection

**Figure 3:**
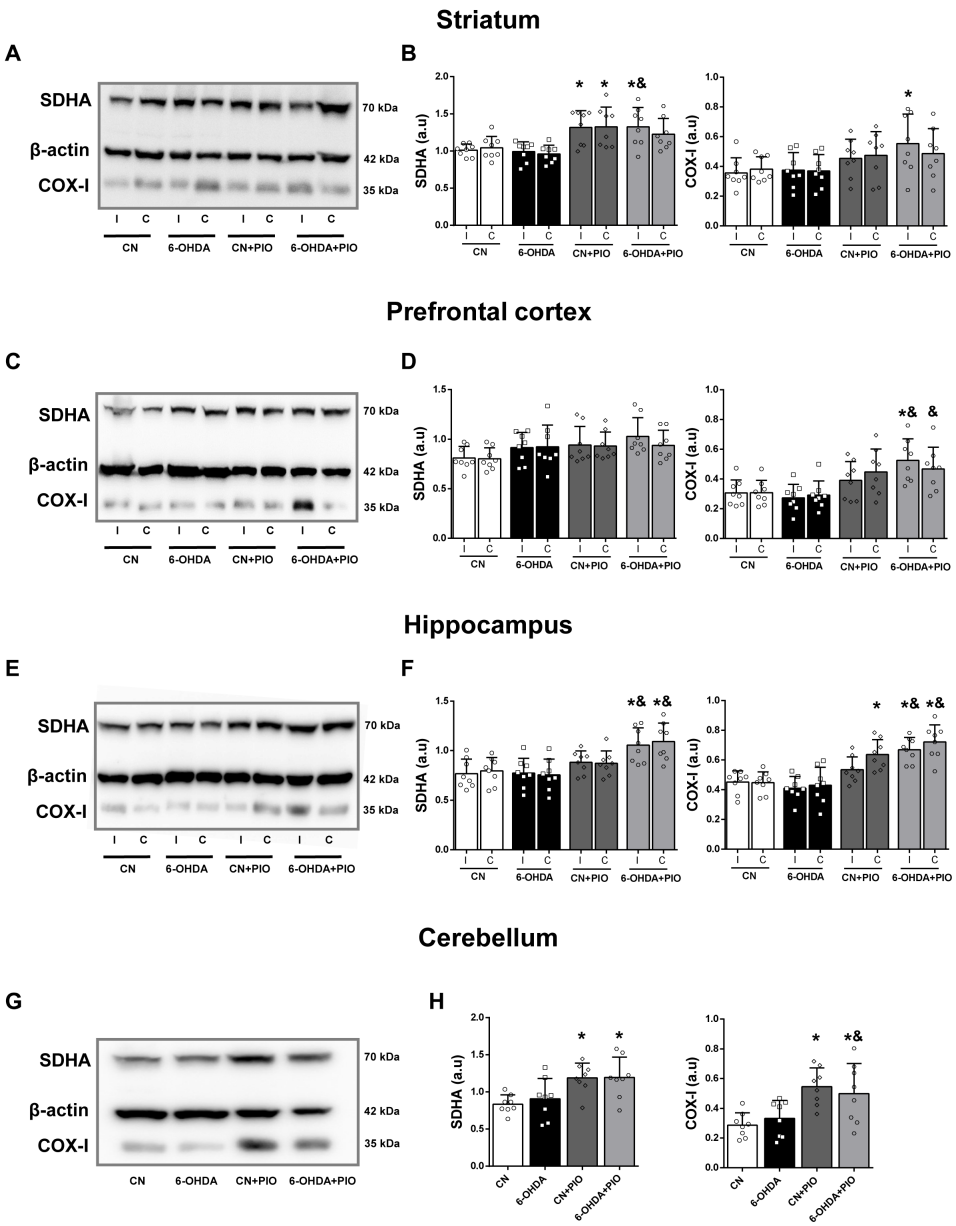
Oral pioglitazone (PIO) enhanced SDHA and COX-I expression levels in multiple brain regions of 6-OHDA-injected and control rats. Representative Western blots **(A,C,E,G)** and quantification **(B,D,F,H)** of SDHA and COX-I protein expression levels in the striatum **(A,B)**, prefrontal cortex **(C,D)**, hippocampus **(E,F)** and cerebellum **(G,H)** of CN, 6-OHDA, CN + PIO and 6-OHDA + PIO groups. β-actin was used as the gel loading control. Results are presented as mean ± SEM of *n* = 8 rats per group. *p < 0.05 vs. CN group and ^&^*p* < 0.05 vs. 6-OHDA group by one-way ANOVA with post hoc Bonferroni tests.

Mounting evidence indicates that mitochondrial dysfunction contributes to the pathogenesis of ADHD (Chang et al., 2020), and PIO can upregulate mitochondrial proteins via PPARγ activation (Bogacka et al., 2005; Ghosh et al., 2007; Strum et al., 2007; Miglio et al., 2009). To examine if PIO treatment influences mitochondrial biogenesis in rats with unilateral striatal lesions, we measured the expression levels of complex IV subunit I (COX-I) (mtDNA-encoded) and the 70 kDa subunit of Complex II (SDHA) (nuclear-encoded) in striatum, prefrontal cortex (PFC), hippocampus and cerebellum by Western blotting. The expression levels of SDHA and COX-I in the striatum did not differ between the 6-OHDA group and CN group on either the I side or C side (Figures 3A,B). However, PIO significantly enhanced SDHA expression in the ipsilateral striatum of 6-OHDA-lesioned rats (6-OHDA + PIO group) compared to both 6-OHDA and CN groups, and also increased COX-I expression in the ipsilateral striatum compared to the CN group (Figures 3A,B). In addition, PIO significantly increased SDHA protein expression in both the ipsilateral and contralateral striatum of CN rats (CN + PIO group), while COX-I expression was not altered (Figures 3A,B). In the PFC, SDHA expression did not differ significantly among groups on either the I or C side (Figures 3C,D). Injection of 6-OHDA tended to reduce COX-I expression in both the ipsilateral and contralateral PFC, but the differences were not statistically significant compared to control rats. Treatment with PIO increased COX-I expression in the ipsilateral PFC of lesioned rats (6-OHDA + PIO group) compared to 6-OHDA and CN groups, and also increased COX-I expression in the contralateral PFC compared to the 6-OHDA group (Figures 3C,D). There was no significant difference in SDHA expression in either the ipsilateral or contralateral hippocampus of 6-OHDA group rats compared to CN group rats (Figures 3E,F), while COX-I was numerically lower in both the ipsilateral and contralateral hippocampus of lesioned rats, but the difference compared to controls did not reach statistical significance. Treatment with PIO significantly increased SDHA and COX-I expression levels in both the ipsilateral and contralateral hippocampus of lesioned rats (6-OHDA + PIO group) compared to 6-OHDA and CN group rats, and also increased expression of COX-I in the contralateral hippocampus of CN rats (CN + PIO group) compared to the CN group (Figures 3E,F). Injection of 6-OHDA into the striatum did not alter cerebellar expression of either SDHA or COX-I compared to the CN group (Figures 3G,H), but PIO significantly increased expression of both proteins in CN rats (CN + PIO group) and lesioned rats (6-OHDA + PIO group) compared to the CN group. Cerebellar COX-I expression was also significantly enhanced in the 6-OHDA + PIO group compared to the 6-OHDA group (Figures 3G,H). Thus, PIO upregulates the expression levels of two major proteins implicated in mitochondrial biogenesis.

**Figure 4:**
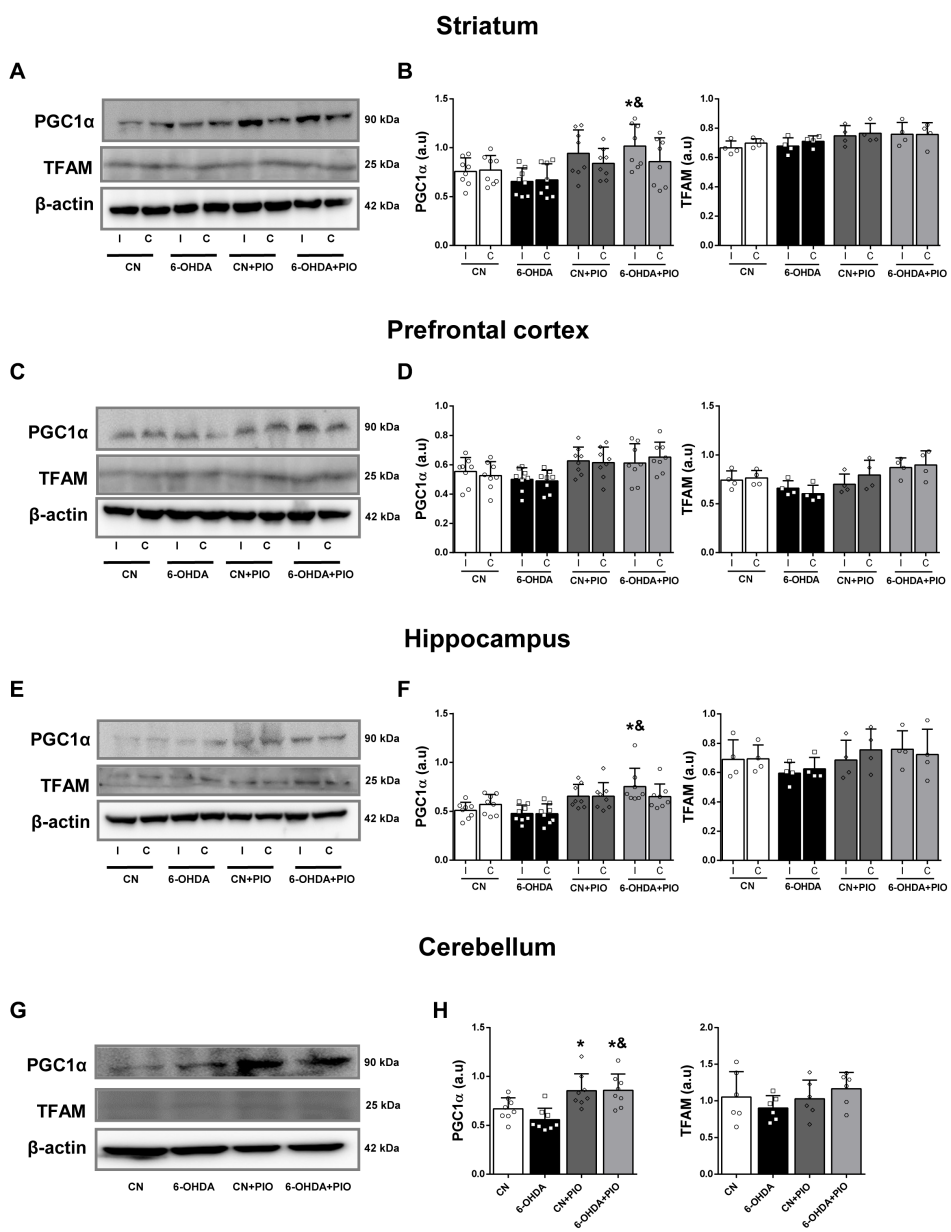
Pioglitazone treatment enhanced PGC1α expression but not TFAM expression in specific brain regions of CN and 6-OHDA rats. Representative Western blots **(A,C,E,G)** and quantification **(B,D,F,H)** of PGC1α and TFAM protein expression in the striatum **(A,B)**, prefrontal cortex **(C,D)**, hippocampus **(E,F)** and cerebellum **(G,H)** of CN, 6-OHDA, CN + PIO and 6-OHDA + PIO groups. β-actin was used as the gel loading control. Results are presented as mean ± SEM of *n* = 8 rats for the PGC1α expression and *n* = 4 rats for TFAM expression, *p < 0.05 vs. CN group and ^&^*p* < 0.05 vs. 6-OHDA group by ANOVA with *post hoc* Bonferroni tests.

In addition to the influence of 6-OHDA and PIO on regional SDHA and COX-I expression levels, we examined effects on expression of PGC1α, considered the master regulator of mitochondrial biogenesis, and TFAM, a downstream transcription factor activated by PGC1α. There were no statistically significant changes in the expression levels of PGC1α and TFAM in either the ipsilateral or contralateral striatum of 6-OHDA group rats compared to the CN group (Figures 4A,B). While striatal 6-OHDA injection tended to reduce PGC1α expression in both the ipsilateral and contralateral striatum, the differences did not reach statistical significance. However, treatment with PIO significantly increased PGC1α expression in the ipsilateral striatum of lesioned rats (6-OHDA + PIO group) compared to the 6-OHDA and CN groups but did not increase PGC1α or TFAM expression in the striatum of CN rats (CN + PIO group; Figures 4A,B). In the PFC, PGC1α and TFAM expression levels did not differ significantly between the 6-OHDA and CN groups (Figures 4C,D). Expression of TFAM tended to be lower in the bilateral PFC of lesioned rats, but the difference from controls did not reach significance. The expression levels of PGC1α and TFAM in bilateral PFC also did not differ significantly following PIO treatment of lesioned and CN rats (CN + PIO and 6-OHDA + PIO groups; Figures 4C,D). In the bilateral hippocampus as well, 6-OHDA did not significantly alter PGC1α or TFAM expression compared to the CN group (Figures 4E,F), although 6-OHDA lesioning did tend to reduce expression of both proteins. Treatment with PIO significantly upregulated PGC1α expression in the ipsilateral hippocampus of lesioned rats (6-OHDA + PIO group) compared to 6-OHDA and CN groups but did not alter the expression levels of either protein in CN rats (CN + PIO group vs. CN group; Figures 4E,F). In the cerebellum, 6-OHDA did not significantly alter PGC1α or TFAM expression compared to the CN group (Figures 4G,H), although the expression levels of both proteins were numerically reduced. Treatment with PIO significantly increased cerebellar expression of PGC1α in CN and lesioned rats (CN + PIO and 6-OHDA + PIO groups) compared to the CN group (Figures 4G,H).

Collectively, unilateral lesioning of the striatum by 6-OHDA injection tended to decrease expression of mitochondrial biogenesis proteins in brain areas associated with the motor and cognitive deficits of ADHD, although these changes did not reach significance. In contrast, PIO promoted expression in multiple brain regions, especially in rats with striatal lesions. Thus, PIO treatment may mitigate deficient mitochondrial biogenesis in ADHD.

### 3.5. Pioglitazone treatment enhanced regional Nrf2 expression

**Figure 5:**
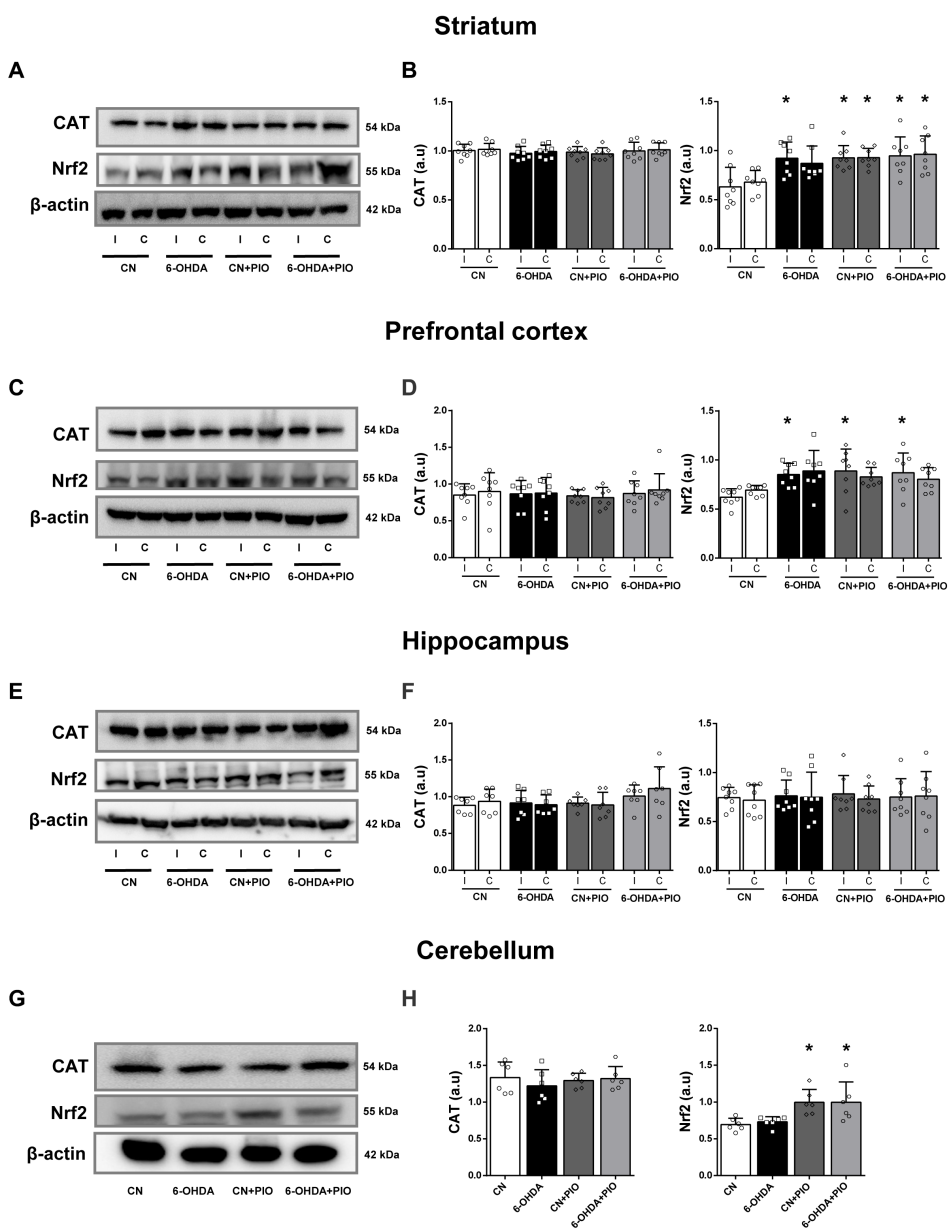
Pioglitazone treatment upregulated Nrf2 but not catalase (CAT) expression in specific brain regions of CN and 6-OHDA group rats. Representative Western blots **(A,C,E,G)** and quantification **(B,D,F,H)** of Nrf2 and CAT proteins in the striatum **(A,B)**, prefrontal cortex **(C,D)**, hippocampus **(E,F)** and cerebellum **(G,H)** of CN, 6-OHDA, CN + PIO and 6-OHDA + PIO groups. β-actin was used as the gel loading control. Results presented as mean ± SEM of *n* = 8 rats per group, *p < 0.05 vs. CN group and ^&^*p* < 0.05 vs. 6-OHDA group by ANOVA with *post hoc* Bonferroni tests.

We further evaluated the effects of 6-OHDA and PIO on expression levels of the constitutive antioxidant enzyme CAT and the phase II transcription factor Nrf2 by Western blotting. The expression of SOD1 was also examined by Western blotting but specific bands were not detected (data not shown). Catalase expression did not differ among groups in either the ipsilateral or contralateral striatum (Figures 5A,B), but 6-OHDA did significantly increase Nrf2 expression in the ipsilateral striatum compared to the CN group (Figures 5A,B). Treatment with PIO enhanced Nrf2 expression in both the ipsilateral and contralateral striatum of lesioned and CN rats (6-OHDA + PIO and CN + PIO groups) compared to the CN group (Figures 5A,B). In the PFC as well, there were no statistically significant differences in CAT expression among groups (Figures 5C,D), but 6-OHDA significantly upregulate ipsilateral Nrf2 expression compared to the CN group. Treatment with PIO also increased Nrf2 expression in the ipsilateral PFC of both lesioned and CN rats (6-OHDA + PIO and CN + PIO groups) compared to the CN group (Figures 5C,D). In hippocampus, there were no statistically significant differences in CAT or Nrf2 expression (Figures 5E,F). In the cerebellum as well, there were no statistically significant differences in CAT expression among groups (Figures 5G,H) and 6-OHDA did not alter the expression of CAT or Nrf2 (Figures 5G,H). However, PIO significantly increased Nrf2 expression in both lesioned and CN rats (6-OHDA + PIO and CN + PIO groups) compared to the CN group. Thus, PIO may enhance phase II detoxification capacity in striatum, PFC and cerebellum of 6-OHDA-lesioned rats, suggesting potential therapeutic utility against ADHD-associated oxidative stress.

## 4. Discussion

Deficits in neural metabolism and catecholaminergic transmission are strongly implicated in ADHD pathogenesis (Prince, 2008; Del Campo et al., 2011), and several experimental animal models with neurotoxin-induced catecholaminergic pathway lesions have been described in recent years with ADHD-like symptoms (Kostrzewa et al., 2016). Among these models, neonatal rodents with 6-OHDA-induced central dopaminergic pathway lesions mimic many key features of the human disorder (Zhang et al., 2002; Bouchatta et al., 2018, 2020). Here we confirm that partial destruction of dopaminergic inputs to the unilateral striatum by 6-OHDA injection recapitulates several neurochemical abnormalities of ADHD and that oral PIO treatment can fully or partially ameliorate these deficits.

Unilateral injection of 6-OHDA into the striatum damaged dopaminergic innervation as evidenced by a 33% decrease in TH expression on the ipsilateral side compared to the contralateral side (Figure 1), consistent with previous studies reporting an approximate 40% reduction of TH expression in the dorsomedial striatum of 6-OHDA-injected rodents (Teicher et al., 1998; Caballero et al., 2011). In addition to damaging dopaminergic terminals in the striatum and nucleus accumbens, 6-OHDA injection can reduce the number of dopaminergic neurons in the substantia nigra through retrograde degeneration (Debeir et al., 2005) and damage dopaminergic projections to the globus pallidus, PFC, cingulate cortex, motor cortex, piriform cortex and hippocampal formation (Debeir et al., 2005; Becker et al., 2018). These extrastriatal effects may explain the structural alterations to the PFC, corpus callosum, cerebellum, amygdala and hippocampus observed in ADHD patients (Arnsten, 2009; Cupertino et al., 2020; Faraone et al., 2021). Collectively, these studies confirm the validity of 6-OHDA-lesioned rats as a model for ADHD pathogenesis and treatment.

In the present study, loss of striatal dopaminergic innervation was associated with motor hyperactivity in the early juvenile stage (PN25), which is also a core clinical feature of ADHD. Similarly, previous studies in rats with neonatal disruption of central dopaminergic pathways by 6-OHDA injection have reported hyperactivity in the juvenile period (e.g., PN25; Zhang et al., 2002; Kostrzewa et al., 2016), again recapitulating clinical ADHD symptoms. However, these same studies also found significantly reduced hyperactivity by adulthood (e.g., PN36 or PN60; Zhang et al., 2002) while we found that hyperactivity persisted at least until PN39. This discrepancy may be attributable to different induction or evaluation protocols. Furthermore, we found no significant effect of PIO on hyperactivity, possibly due to an insufficient dose or treatment duration as higher doses for longer periods have been shown to influence the behavioral phenotypes of other disease models. For instance, administration of 10 and 20 mg/kg PIO orally for 27 days attenuated social impairments, repetitive behaviors, hyperactivity, anxiety and low exploratory activity in an animal model of autism (Mirza and Sharma, 2019a,b). Likewise, 10, 20 and 40 mg/kg PIO for 21 days improved locomotor activity, beam walk performance and rotarod performance in a Huntington’s disease model established by quinolinic acid treatment (Kalonia et al., 2010). Similarly, 30 mg/kg PIO but not 10 mg/kg for 14 days improved cognitive performance and lowered oxidative stress in rats with streptozotocin-induced diabetes (Pathan et al., 2006). Notably, 30 or 60 mg PIO orally for 3–4 months also effectively improved ADHD symptoms in a small cohort of pediatric patients (Boris et al., 2007), while a 10-week, randomized, double-blind, parallel-group, placebo-controlled clinical trial of children with autism found that 30 mg daily PIO as adjunctive treatment with risperidone improved hyperactivity, irritability and lethargy (Ghaleiha et al., 2015). Thus, a higher-dose or longer PIO treatment regimen may improve ADHD-like symptoms as well.

Mitochondrial dysfunction contributes to a variety of neurodegenerative and neurodevelopmental disorders, including ADHD, suggesting that mitochondria are a promising therapeutic target (Li et al., 2017; Ogutlu et al., 2020; Giannoulis et al., 2022). Indeed, several recent studies have found that induction of mitochondrial biogenesis using PIO can mitigate abnormalities in various neurological disorder models (Miglio et al., 2009; Corona and Duchen, 2016; Li et al., 2017). In the current study, PIO treatment increased the expression levels of COX-I and SDHA, both of which are required for mitochondrial biogenesis, in striatum, cerebellum and hippocampus of both lesioned (6-OHDA-injected) and control (CN group) rats (Figure 3), and also upregulated COX-I (but not SDHA) expression in the PFC of lesioned and CN rats. Additionally, PIO increased PGC1α expression in the striatum, hippocampus and cerebellum of 6-OHDA group rats as well as in the cerebellum of CN group rats (Figure 4). In contrast, no change in PGC1α expression was observed in the PFC of either lesioned or CN rats and TFAM expression was unchanged in all brain regions analyzed. Pioglitazone treatment at a higher concentration or for a longer time may upregulate expression of mitochondrial biogenesis-associated proteins in these regions as well. For instance, a 4-week treatment with 10 mg/kg PIO increased PGC1α expression in a diabetic rat model (Mostafa Tork et al., 2019), 20 mg/kg daily for 7 days partially rescued mitochondrial dysfunction in the striatum and substantia nigra of lipopolysaccharide-treated rats (Hunter et al., 2007), and 10 mg/kg/day PIO for 3 months upregulated mitochondrial biogenesis in spontaneously diabetic Goto-Kakizaki rats (Shen et al., 2008).

Deficits in neural antioxidant capacity may also contribute to ADHD development. Dopamine and noradrenaline are easily auto-oxidized and so may induce oxidative stress, mitochondrial dysfunction and neuronal damage under conditions of reduced antioxidant capacity (Napolitano et al., 2011; Goldstein et al., 2014; Alvarez-Arellano et al., 2020; Corona, 2020). In fact, reduced expression levels of SOD, glutathione-S-transferase, GPx, glutathione (GSH), and CAT have been reported in children with ADHD (Selek et al., 2008; Nasim et al., 2019). Moreover, the drugs used to treat ADHD may cause the accumulation of catecholamines, leading to enhanced autoxidation and oxidative stress (Martins et al., 2006; Corona, 2020). For instance, MPH administration reduced SOD, GSH, GPx and glutathione reductase activities in rats, resulting in oxidative stress and neurodegeneration (Motaghinejad et al., 2017; Dutt et al., 2020). Alternatively, PIO has been shown to reduce oxidative stress by upregulating antioxidant pathways (Corona and Duchen, 2016). In this study, however, PIO did not induce CAT expression in any of the analyzed brain regions (Figure 5). Similarly, treatment with 10 and 30 mg/kg PIO for 4 weeks did not alter the levels of SOD, CAT and GSH in renal tissue of diabetic model rats (Kuru Karabas et al., 2013). However, it is still critical to assess the effects of PIO on local antioxidant activity and resistance to oxidative stress as average expression or activity levels in large tissue samples may not reflect intracellular antioxidant and neuroprotective capacities. For instance, PIO has been reported to increase CAT activity but not expression (Kalonia et al., 2011; Beheshti et al., 2019).

Pioglitazone has also been reported to activate Nrf2 and downstream pathways in substantia nigra, striatum, hippocampus, PFC, cerebellum and brainstem (Rafati et al., 2015; Mirza and Sharma, 2019a; Blackburn et al., 2022). For instance, treatment with 30 mg/kg PIO for 7 days reduced LPS-induced dopaminergic neuronal loss and enhanced Nrf2 mRNA expression in mouse (Zakaria et al., 2019). Also, intracerebroventricular injection of PIO for 5 days at 6 mmol/L increased expression of Nrf2 and its target HO-1 in the penumbra surrounding ischemic brain tissue of rats (Zhao et al., 2021). Similarly, we found that PIO treatment significantly increased Nrf2 expression in the ipsilateral striatum, PFC and cerebellum of both lesioned and CN rats, although not in the hippocampus (Figure 5). Moreover, increased Nrf2 expression was observed in the striatum and PFC of 6-OHDA group rats (Figure 5), suggesting that Nrf2 upregulation is a compensatory protective response to oxidative stress caused by 6-OHDA (Heikkila and Cohen, 1973; Glinka et al., 1997). We suggest that this response may be augmented by PIO for more effective neuroprotection.

### 4.1. Limitations of the study

The experimental rodent models of ADHD are heterogeneous in the pathophysiological alterations, on the ability to mimic behavioral symptoms (hyperactivity, inattention and impulsivity) and the response to pharmacological treatments. Thus, the existing experimental models of ADHD cannot represent all the different ADHD subtypes or altered gene expression (highly polygenic predisposition to ADHD) (Russell, 2011; Regan et al., 2022). Therefore, the validation of experimental rodent models is complicated and there is no ideal animal model of ADHD that can recapitulate all the core symptoms. Since most of the underlying aetiology of ADHD is still unknown, it is main to emphasize that experimental rodent models to study ADHD are evaluated with the following criteria: face validity, construct validity and predictive validity (Russell, 2011). The construct validity of presumed animal models of ADHD would always be limited (Sontag et al., 2010). Nevertheless, it is imperative to mark that ADHD is commonly associated with dysfunction in the dopaminergic system (Prince, 2008; Del Campo et al., 2011). Consequently, construct validity in animal models of ADHD can be established, in some measure, by demonstrating alterations of the dopaminergic system. However, animal models cannot truly reflect the human disorder, even so, can provide insight into the possible mechanism involved in the pathogenesis of disease and for studying some of the molecular, genetic and cellular events related to ADHD, also can provide insight into alterations on several brain areas, individual tissues, circuits and neurotransmitter systems and to find new therapeutic targets. Due to deficits in the dopaminergic neurotransmission in patients with ADHD, the neonatal 6-OHDA-lesioned rodents as experimental models to study ADHD-like behaviors have been used, such as the neonate rat lesioned unilaterally into the striatum with the neurotoxin 6-OHDA (Caballero et al., 2011) however, this model has certain limitations that influenced the results obtained. The limitations of the study were the absence of measurement of dopaminergic innervation and the dopamine levels associated with the 6-OHDA injection; though, the decrease in TH immunoreactivity observed can relate as an indirect measure of dopamine depletion in the striatum. In addition, 6-OHDA injection reduced the number of dopaminergic neurons in the substantia nigra through retrograde degeneration (Debeir et al., 2005). Thus, this model can also exemplify that the 6-OHDA-lesion could elicit dopamine depletion and is sufficient to produce some ADHD-like behaviors and hence have implications for research on ADHD (Teicher et al., 1998; Kostrzewa et al., 2016). Moreover, throughout the literature, a difference in 6-OHDA dose, volumes of 6-OHDA injected, and the site of injection of 6-OHDA (injected intracisternal or into the lateral ventricle, also used) (Zhang et al., 2002; Bouchatta et al., 2018) and have an impact on the rate of neurotoxin uptake and is critical to the predictive validity since depending on the administration route may be variations in the result on the behavior response and the response to treatment with different medications or compounds. Therefore, a comprehensive assessment of ADHD-like symptoms is still missing, and data from several animal models remain largely unavailable.

## 5. Conclusion

Our findings suggest that treatment with PIO for 14 days can increase mitochondrial biogenesis and phase II detoxification pathways in an experimental rat model of ADHD, thereby protecting against neuronal damage. In contrast, this dose regimen is insufficient for upregulating catalase expression or suppressing behavioral hyperactivity. Comprehensive studies are needed to identify the optimal PIO dose for mitigation of ADHD-like behavioral symptoms and induction of endogenous neuroprotective mechanisms, either when applied alone or in combination with drugs currently used for ADHD treatment. Clinical trials and well-designed prospective studies are also warranted to examine the therapeutic potential of PIO as an adjuvant treatment against the mitochondrial dysfunction and oxidative stress observed in ADHD patients.

## Data availability statement

The original contributions presented in the study are included in the article/supplementary material, further inquiries can be directed to the corresponding author.

## Ethics statement

The animal study was reviewed and approved by Hospital Infantil de Mexico Federico Gómez, Institutional Ethical, Animal Care and Use Committees.

## Author contributions

DV-G performed the experiments and analyzed the data. JCC designed the study and wrote the manuscript. All authors contributed to the article and approved the submitted version.

## Funding

This work was supported by Fondos Federales HIM 2018-030 SSA 1497. DV-G was recipient of a fellowship from CONACYT.

## Conflict of interest

The authors declare that the research was conducted in the absence of any commercial or financial relationships that could be construed as a potential conflict of interest.

## Publisher’s note

All claims expressed in this article are solely those of the authors and do not necessarily represent those of their affiliated organizations, or those of the publisher, the editors and the reviewers. Any product that may be evaluated in this article, or claim that may be made by its manufacturer, is not guaranteed or endorsed by the publisher.

## References

[ref1] Abdallah D. M. (2010). Anticonvulsant potential of the peroxisome proliferator-activated receptor gamma agonist pioglitazone in pentylenetetrazole-induced acute seizures and kindling in mice. Brain Res. 1351, 246–253. doi: 10.1016/j.brainres.2010.06.034, PMID: 20599832

[ref2] Alvarez-Arellano L. Gonzalez-Garcia N. Salazar-Garcia M. Corona J. C. (2020). Antioxidants as a potential target against inflammation and oxidative stress in attention-deficit/hyperactivity disorder. Antioxidants 9. doi: 10.3390/antiox9020176, PMID: 32098021 PMC7070894

[ref3] Arnsten A. F. (2009). Toward a new understanding of attention-deficit hyperactivity disorder pathophysiology: an important role for prefrontal cortex dysfunction. CNS Drugs 23, 33–41. doi: 10.2165/00023210-200923000-00005 19621976

[ref4] Becker B. Demirbas M. Johann S. Zendedel A. Beyer C. Clusmann H.. (2018). Effect of intrastriatal 6-OHDA lesions on extrastriatal brain structures in the mouse. Mol. Neurobiol. 55, 4240–4252. doi: 10.1007/s12035-017-0637-9, PMID: 28616718

[ref5] Beheshti F. Hosseini M. Hashemzehi M. Soukhtanloo M. Khazaei M. Shafei M. N. (2019). The effects of PPAR-gamma agonist pioglitazone on hippocampal cytokines, brain-derived neurotrophic factor, memory impairment, and oxidative stress status in lipopolysaccharide-treated rats. Iran. J. Basic Med. Sci. 22, 940–948. doi: 10.22038/ijbms.2019.36165.8616, PMID: 31579451 PMC6760489

[ref6] Blackburn J. K. Jamwal S. Wang W. Elsworth J. D. (2022). Pioglitazone transiently stimulates paraoxonase-2 expression in male nonhuman primate brain: implications for sex-specific therapeutics in neurodegenerative disorders. Neurochem. Int. 152:105222. doi: 10.1016/j.neuint.2021.105222, PMID: 34767873 PMC8712400

[ref7] Bogacka I. Xie H. Bray G. A. Smith S. R. (2005). Pioglitazone induces mitochondrial biogenesis in human subcutaneous adipose tissue in vivo. Diabetes 54, 1392–1399. doi: 10.2337/diabetes.54.5.1392, PMID: 15855325

[ref8] Boris M. Kaiser C. C. Goldblatt A. Elice M. W. Edelson S. M. Adams J. B.. (2007). Effect of pioglitazone treatment on behavioral symptoms in autistic children. J. Neuroinflammation 4:3. doi: 10.1186/1742-2094-4-3, PMID: 17207275 PMC1781426

[ref9] Bouchatta O. Manouze H. Ba-M’Hamed S. Landry M. Bennis M. (2020). Neonatal 6-OHDA lesion model in mouse induces cognitive dysfunctions of attention-deficit/hyperactivity disorder (ADHD) during young age. Front. Behav. Neurosci. 14:27. doi: 10.3389/fnbeh.2020.00027, PMID: 32174817 PMC7054716

[ref10] Bouchatta O. Manouze H. Bouali-Benazzouz R. Kerekes N. Ba-M'hamed S. Fossat P.. (2018). Neonatal 6-OHDA lesion model in mouse induces attention-deficit/ hyperactivity disorder (ADHD)-like behaviour. Sci. Rep. 8:15349. doi: 10.1038/s41598-018-33778-0, PMID: 30337626 PMC6193955

[ref11] Caballero M. Nunez F. Ahern S. Cuffi M. L. Carbonell L. Sanchez S.. (2011). Caffeine improves attention deficit in neonatal 6-OHDA lesioned rats, an animal model of attention deficit hyperactivity disorder (ADHD). Neurosci. Lett. 494, 44–48. doi: 10.1016/j.neulet.2011.02.050, PMID: 21362462

[ref12] Chang X. Liu Y. Mentch F. Glessner J. Qu H. Nguyen K.. (2020). Mitochondrial DNA haplogroups and risk of attention deficit and hyperactivity disorder in European Americans. Transl. Psychiatry 10:370. doi: 10.1038/s41398-020-01064-1, PMID: 33139694 PMC7608630

[ref13] Chen T. Jin X. Crawford B. H. Cheng H. Saafir T. B. Wagner M. B.. (2012). Cardioprotection from oxidative stress in the newborn heart by activation of PPARgamma is mediated by catalase. Free Radic. Biol. Med. 53, 208–215. doi: 10.1016/j.freeradbiomed.2012.05.014, PMID: 22609424

[ref14] Chung S. S. Kim M. Youn B. S. Lee N. S. Park J. W. Lee I. K.. (2009). Glutathione peroxidase 3 mediates the antioxidant effect of peroxisome proliferator-activated receptor gamma in human skeletal muscle cells. Mol. Cell. Biol. 29, 20–30. doi: 10.1128/MCB.00544-08, PMID: 18936159 PMC2612482

[ref15] Clemow D. B. (2017). Misuse of methylphenidate. Curr. Top. Behav. Neurosci. 34, 99–124. doi: 10.1007/7854_2015_426 26695166

[ref16] Corona J. C. (2018). Natural compounds for the Management of Parkinson’s disease and attention-deficit/hyperactivity disorder. Biomed. Res. Int. 2018:4067597. doi: 10.1155/2018/4067597, PMID: 30596091 PMC6282143

[ref17] Corona J. C. (2020). Role of oxidative stress and neuroinflammation in attention-deficit/hyperactivity disorder. Antioxidants 9. doi: 10.3390/antiox9111039, PMID: 33114154 PMC7690797

[ref18] Corona J. C. (2021). “Pharmacological approaches for the treatment of attention-deficit/hyperactivity disorder” in Attention-deficit hyperactivity disorder: diagnosis, prevalence and treatment. ed. Kyser B. M. (New York: Nova Science Publishers, Inc), 1–39.

[ref19] Corona J. C. de Souza S. C. Duchen M. R. (2014). PPARgamma activation rescues mitochondrial function from inhibition of complex I and loss of PINK1. Exp. Neurol. 253, 16–27. doi: 10.1016/j.expneurol.2013.12.012, PMID: 24374061

[ref20] Corona J. C. Duchen M. R. (2015a). Impaired mitochondrial homeostasis and neurodegeneration: towards new therapeutic targets? J. Bioenerg. Biomembr. 47, 89–99. doi: 10.1007/s10863-014-9576-6, PMID: 25216534 PMC4323516

[ref21] Corona J. C. Duchen M. R. (2015b). PPARgamma and PGC-1alpha as therapeutic targets in Parkinson’s. Neurochem. Res. 40, 308–316. doi: 10.1007/s11064-014-1377-0, PMID: 25007880 PMC4326663

[ref22] Corona J. C. Duchen M. R. (2016). PPARgamma as a therapeutic target to rescue mitochondrial function in neurological disease. Free Radic. Biol. Med. 100, 153–163. doi: 10.1016/j.freeradbiomed.2016.06.023, PMID: 27352979 PMC5145801

[ref23] Cortese S. (2020). Pharmacologic treatment of attention deficit-hyperactivity disorder. N. Engl. J. Med. 383, 1050–1056. doi: 10.1056/NEJMra1917069 32905677

[ref24] Cupertino R. B. Soheili-Nezhad S. Grevet E. H. Bandeira C. E. Picon F. A. Tavares M. E. A.. (2020). Reduced fronto-striatal volume in attention-deficit/hyperactivity disorder in two cohorts across the lifespan. Neuroimage Clin. 28:102403. doi: 10.1016/j.nicl.2020.102403, PMID: 32949876 PMC7502360

[ref25] Debeir T. Ginestet L. Francois C. Laurens S. Martel J. C. Chopin P.. (2005). Effect of intrastriatal 6-OHDA lesion on dopaminergic innervation of the rat cortex and globus pallidus. Exp. Neurol. 193, 444–454. doi: 10.1016/j.expneurol.2005.01.007, PMID: 15869947

[ref26] Del Campo N. Chamberlain S. R. Sahakian B. J. Robbins T. W. (2011). The roles of dopamine and noradrenaline in the pathophysiology and treatment of attention-deficit/hyperactivity disorder. Biol. Psychiatry 69, e145–e157. doi: 10.1016/j.biopsych.2011.02.036, PMID: 21550021

[ref27] Dutt M. Dharavath R. N. Kaur T. Chopra K. Sharma S. (2020). Differential effects of alprazolam against methylphenidate-induced neurobehavioral alterations. Physiol. Behav. 222:112935. doi: 10.1016/j.physbeh.2020.112935, PMID: 32413536

[ref28] El-Sayed K. Ali D. A. Maher S. A. Ghareeb D. Selim S. Albogami S.. (2022). Prophylactic and ameliorative effects of PPAR-gamma agonist pioglitazone in improving oxidative stress, germ cell apoptosis and inflammation in gentamycin-induced testicular damage in adult male albino rats. Antioxidants 11. doi: 10.3390/antiox11020191, PMID: 35204074 PMC8868260

[ref29] Emmerzaal T. L. Nijkamp G. Veldic M. Rahman S. Andreazza A. C. Morava E.. (2021). Effect of neuropsychiatric medications on mitochondrial function: for better or for worse. Neurosci. Biobehav. Rev. 127, 555–571. doi: 10.1016/j.neubiorev.2021.05.001 34000348

[ref30] Faraone S. V. Banaschewski T. Coghill D. Zheng Y. Biederman J. Bellgrove M. A.. (2021). The world federation of ADHD international consensus statement: 208 evidence-based conclusions about the disorder. Neurosci. Biobehav. Rev. 128, 789–818. doi: 10.1016/j.neubiorev.2021.01.022, PMID: 33549739 PMC8328933

[ref31] Forman H. J. Zhang H. (2021). Targeting oxidative stress in disease: promise and limitations of antioxidant therapy. Nat. Rev. Drug Discov. 20, 689–709. doi: 10.1038/s41573-021-00233-1, PMID: 34194012 PMC8243062

[ref32] Ghaleiha A. Rasa S. M. Nikoo M. Farokhnia M. Mohammadi M. R. Akhondzadeh S. (2015). A pilot double-blind placebo-controlled trial of pioglitazone as adjunctive treatment to risperidone: effects on aberrant behavior in children with autism. Psychiatry Res. 229, 181–187. doi: 10.1016/j.psychres.2015.07.043, PMID: 26208985

[ref33] Ghosh S. Patel N. Rahn D. McAllister J. Sadeghi S. Horwitz G.. (2007). The thiazolidinedione pioglitazone alters mitochondrial function in human neuron-like cells. Mol. Pharmacol. 71, 1695–1702. doi: 10.1124/mol.106.033845, PMID: 17387142

[ref34] Giannoulis S. V. Muller D. Kennedy J. L. Goncalves V. (2022). Systematic review of mitochondrial genetic variation in attention-deficit/hyperactivity disorder. Eur. Child Adolesc. Psychiatry. doi: 10.1007/s00787-022-02030-6, PMID: 35796884

[ref35] Girnun G. D. Domann F. E. Moore S. A. Robbins M. E. (2002). Identification of a functional peroxisome proliferator-activated receptor response element in the rat catalase promoter. Mol. Endocrinol. 16, 2793–2801. doi: 10.1210/me.2002-0020, PMID: 12456800

[ref36] Glinka Y. Gassen M. Youdim M. B. (1997). Mechanism of 6-hydroxydopamine neurotoxicity. J. Neural Transm. Suppl. 50, 55–66. doi: 10.1007/978-3-7091-6842-4_7 9120425

[ref37] Gold M. S. Blum K. Oscar-Berman M. Braverman E. R. (2014). Low dopamine function in attention deficit/hyperactivity disorder: should genotyping signify early diagnosis in children? Postgrad. Med. 126, 153–177. doi: 10.3810/pgm.2014.01.2735, PMID: 24393762 PMC4074363

[ref38] Goldstein D. S. Kopin I. J. Sharabi Y. (2014). Catecholamine autotoxicity. Implications for pharmacology and therapeutics of Parkinson disease and related disorders. Pharmacol. Ther. 144, 268–282. doi: 10.1016/j.pharmthera.2014.06.006, PMID: 24945828 PMC4591072

[ref39] Gomes K. M. Petronilho F. C. Mantovani M. Garbelotto T. Boeck C. R. Dal-Pizzol F.. (2008). Antioxidant enzyme activities following acute or chronic methylphenidate treatment in young rats. Neurochem. Res. 33, 1024–1027. doi: 10.1007/s11064-007-9544-1 18049893

[ref40] Gray J. D. Punsoni M. Tabori N. E. Melton J. T. Fanslow V. Ward M. J.. (2007). Methylphenidate administration to juvenile rats alters brain areas involved in cognition, motivated behaviors, appetite, and stress. J. Neurosci. 27, 7196–7207. doi: 10.1523/JNEUROSCI.0109-07.2007, PMID: 17611273 PMC6794586

[ref41] Heikkila R. E. Cohen G. (1973). 6-Hydroxydopamine: evidence for superoxide radical as an oxidative intermediate. Science 181, 456–457. doi: 10.1126/science.181.4098.456, PMID: 4718113

[ref42] Hunter R. L. Dragicevic N. Seifert K. Choi D. Y. Liu M. Kim H. C.. (2007). Inflammation induces mitochondrial dysfunction and dopaminergic neurodegeneration in the nigrostriatal system. J. Neurochem. 100, 1375–1386. doi: 10.1111/j.1471-4159.2006.04327.x 17254027

[ref43] Jamwal S. Blackburn J. K. Elsworth J. D. (2021). PPARgamma/PGC1alpha signaling as a potential therapeutic target for mitochondrial biogenesis in neurodegenerative disorders. Pharmacol. Ther. 219:107705. doi: 10.1016/j.pharmthera.2020.107705, PMID: 33039420 PMC7887032

[ref44] Kalonia H. Kumar P. Kumar A. (2010). Pioglitazone ameliorates behavioral, biochemical and cellular alterations in quinolinic acid induced neurotoxicity: possible role of peroxisome proliferator activated receptor-upsilon (PPARUpsilon) in Huntington’s disease. Pharmacol. Biochem. Behav. 96, 115–124. doi: 10.1016/j.pbb.2010.04.018 20450929

[ref45] Kalonia H. Kumar P. Kumar A. (2011). Attenuation of proinflammatory cytokines and apoptotic process by verapamil and diltiazem against quinolinic acid induced Huntington like alterations in rats. Brain Res. 1372, 115–126. doi: 10.1016/j.brainres.2010.11.060, PMID: 21112316

[ref46] Koda K. Ago Y. Cong Y. Kita Y. Takuma K. Matsuda T. (2010). Effects of acute and chronic administration of atomoxetine and methylphenidate on extracellular levels of noradrenaline, dopamine and serotonin in the prefrontal cortex and striatum of mice. J. Neurochem. 114, 259–270. doi: 10.1111/j.1471-4159.2010.06750.x, PMID: 20403082

[ref47] Kostrzewa J. P. Kostrzewa R. A. Kostrzewa R. M. Brus R. Nowak P. (2016). Perinatal 6-hydroxydopamine modeling of ADHD. Curr. Top. Behav. Neurosci. 29, 279–293. doi: 10.1007/7854_2015_397, PMID: 26475157

[ref48] Kuru Karabas M. Ayhan M. Guney E. Serter M. Meteoglu I. (2013). The effect of pioglitazone on antioxidant levels and renal histopathology in streptozotocin-induced diabetic rats. ISRN Endocrinol 2013:858690. doi: 10.1155/2013/858690, PMID: 23762597 PMC3665254

[ref49] Lee C. (2017). Collaborative power of Nrf2 and PPARgamma activators against metabolic and drug-induced oxidative injury. Oxidative Med. Cell. Longev. 2017:1378175. doi: 10.1155/2017/1378175, PMID: 28928902 PMC5591982

[ref50] Li P. A. Hou X. Hao S. (2017). Mitochondrial biogenesis in neurodegeneration. J. Neurosci. Res. 95, 2025–2029. doi: 10.1002/jnr.24042 28301064

[ref51] Martins M. R. Reinke A. Petronilho F. C. Gomes K. M. Dal-Pizzol F. Quevedo J. (2006). Methylphenidate treatment induces oxidative stress in young rat brain. Brain Res. 1078, 189–197. doi: 10.1016/j.brainres.2006.01.004, PMID: 16494852

[ref52] Miglio G. Rosa A. C. Rattazzi L. Collino M. Lombardi G. Fantozzi R. (2009). PPARgamma stimulation promotes mitochondrial biogenesis and prevents glucose deprivation-induced neuronal cell loss. Neurochem. Int. 55, 496–504. doi: 10.1016/j.neuint.2009.05.001, PMID: 19442697

[ref53] Mirza R. Sharma B. (2019a). Beneficial effects of pioglitazone, a selective peroxisome proliferator-activated receptor-gamma agonist in prenatal valproic acid-induced behavioral and biochemical autistic like features in Wistar rats. Int. J. Dev. Neurosci. 76, 6–16. doi: 10.1016/j.ijdevneu.2019.05.006 31128204

[ref54] Mirza R. Sharma B. (2019b). A selective peroxisome proliferator-activated receptor-gamma agonist benefited propionic acid induced autism-like behavioral phenotypes in rats by attenuation of neuroinflammation and oxidative stress. Chem. Biol. Interact. 311:108758. doi: 10.1016/j.cbi.2019.108758, PMID: 31348919

[ref55] Morton W. A. Stockton G. G. (2000). Methylphenidate abuse and psychiatric side effects. Prim Care Companion J. Clin. Psychiatry 2, 159–164. doi: 10.4088/PCC.v02n0502, PMID: 15014637 PMC181133

[ref56] Mostafa Tork O. Ahmed Rashed L. Bakr Sadek N. Abdel-Tawab M. S. (2019). Targeting altered mitochondrial biogenesis in the brain of diabetic rats: potential effect of pioglitazone and Exendin-4. Rep. Biochem. Mol. Biol. 8, 287–300. PMID: 32274400 PMC7103073

[ref57] Motaghinejad M. Motevalian M. Shabab B. Fatima S. (2017). Effects of acute doses of methylphenidate on inflammation and oxidative stress in isolated hippocampus and cerebral cortex of adult rats. J. Neural Transm. 124, 121–131. doi: 10.1007/s00702-016-1623-5, PMID: 27682635

[ref58] Napolitano A. Manini P. d'Ischia M. (2011). Oxidation chemistry of catecholamines and neuronal degeneration: an update. Curr. Med. Chem. 18, 1832–1845. doi: 10.2174/092986711795496863, PMID: 21466469

[ref59] Nasim S. Naeini A. A. Najafi M. Ghazvini M. Hassanzadeh A. (2019). Relationship between antioxidant status and attention deficit hyperactivity disorder among children. Int. J. Prev. Med. 10:41. doi: 10.4103/ijpvm.IJPVM_80_18, PMID: 31057726 PMC6484508

[ref60] Nolfi-Donegan D. Braganza A. Shiva S. (2020). Mitochondrial electron transport chain: oxidative phosphorylation, oxidant production, and methods of measurement. Redox Biol. 37:101674. doi: 10.1016/j.redox.2020.101674, PMID: 32811789 PMC7767752

[ref61] Ogutlu H. Esin I. S. Erdem H. B. Tatar A. Dursun O. B. (2020). Mitochondrial DNA copy number is associated with attention deficit hyperactivity disorder. Psychiatr. Danub. 32, 168–175. doi: 10.24869/psyd.2020.168 32796781

[ref62] Pathan A. R. Viswanad B. Sonkusare S. K. Ramarao P. (2006). Chronic administration of pioglitazone attenuates intracerebroventricular streptozotocin induced-memory impairment in rats. Life Sci. 79, 2209–2216. doi: 10.1016/j.lfs.2006.07.018, PMID: 16904700

[ref63] Picca A. Calvani R. Coelho-Junior H. J. Landi F. Bernabei R. Marzetti E. (2020). Mitochondrial dysfunction, oxidative stress, and neuroinflammation: intertwined roads to neurodegeneration. Antioxidants 9. doi: 10.3390/antiox9080647, PMID: 32707949 PMC7466131

[ref64] Posner J. Polanczyk G. V. Sonuga-Barke E. (2020). Attention-deficit hyperactivity disorder. Lancet 395, 450–462. doi: 10.1016/S0140-6736(19)33004-1, PMID: 31982036 PMC7880081

[ref65] Prince J. (2008). Catecholamine dysfunction in attention-deficit/hyperactivity disorder: an update. J. Clin. Psychopharmacol. 28, S39–S45. doi: 10.1097/JCP.0b013e318174f92a 18480676

[ref66] Rafati A. Yazdani H. Noorafshan A. (2015). Pioglitazone ameliorates neuron loss in the cortex after aluminum-treatment in rats. Neurol. Res. Int. 2015, 381934–381938. doi: 10.1155/2015/381934, PMID: 26167300 PMC4475701

[ref67] Regan S. L. Williams M. T. Vorhees C. V. (2022). Review of rodent models of attention deficit hyperactivity disorder. Neurosci. Biobehav. Rev. 132, 621–637. doi: 10.1016/j.neubiorev.2021.11.041, PMID: 34848247 PMC8816876

[ref68] Rey F. Ottolenghi S. Zuccotti G. V. Samaja M. Carelli S. (2022). Mitochondrial dysfunctions in neurodegenerative diseases: role in disease pathogenesis, strategies for analysis and therapeutic prospects. Neural Regen. Res. 17, 754–758. doi: 10.4103/1673-5374.322430, PMID: 34472461 PMC8530118

[ref69] Russell V. A. (2011). Overview of animal models of attention deficit hyperactivity disorder (ADHD). Curr. Protoc. Neurosci. 9:Unit 9.35. doi: 10.1002/0471142301.ns0935s54 21207367

[ref70] Selek S. Savas H. A. Gergerlioglu H. S. Bulut M. Yilmaz H. R. (2008). Oxidative imbalance in adult attention deficit/hyperactivity disorder. Biol. Psychol. 79, 256–259. doi: 10.1016/j.biopsycho.2008.06.005, PMID: 18644422

[ref71] Shellenberg T. P. Stoops W. W. Lile J. A. Rush C. R. (2020). An update on the clinical pharmacology of methylphenidate: therapeutic efficacy, abuse potential and future considerations. Expert. Rev. Clin. Pharmacol. 13, 825–833. doi: 10.1080/17512433.2020.1796636, PMID: 32715789

[ref72] Shen W. Hao J. Tian C. Ren J. Yang L. Li X.. (2008). A combination of nutriments improves mitochondrial biogenesis and function in skeletal muscle of type 2 diabetic Goto-Kakizaki rats. PLoS One 3:e2328. doi: 10.1371/journal.pone.0002328, PMID: 18523557 PMC2391295

[ref73] Sontag T. A. Tucha O. Walitza S. Lange K. W. (2010). Animal models of attention deficit/hyperactivity disorder (ADHD): a critical review. Atten. Defic. Hyperact. Disord. 2, 1–20. doi: 10.1007/s12402-010-0019-x 21432586

[ref74] Strum J. C. Shehee R. Virley D. Richardson J. Mattie M. Selley P.. (2007). Rosiglitazone induces mitochondrial biogenesis in mouse brain. J. Alzheimers Dis. 11, 45–51. doi: 10.3233/jad-2007-11108, PMID: 17361034

[ref75] Teicher M. H. Andersen S. L. Campbell A. Gelbard H. A. Baldessarini R. J. (1998). Progressive accumbens degeneration after neonatal striatal 6-hydroxydopamine in rats. Neurosci. Lett. 247, 99–102. doi: 10.1016/s0304-3940(98)00281-x, PMID: 9655602

[ref76] Ulusoy G. K. Celik T. Kayir H. Gursoy M. Isik A. T. Uzbay T. I. (2011). Effects of pioglitazone and retinoic acid in a rotenone model of Parkinson’s disease. Brain Res. Bull. 85, 380–384. doi: 10.1016/j.brainresbull.2011.05.001 21600965

[ref77] Verma P. Singh A. Nthenge-Ngumbau D. N. Rajamma U. Sinha S. Mukhopadhyay K.. (2016). Attention deficit-hyperactivity disorder suffers from mitochondrial dysfunction. BBA Clin. 6, 153–158. doi: 10.1016/j.bbacli.2016.10.003, PMID: 27896136 PMC5121149

[ref78] Zakaria A. Rady M. Mahran L. Abou-Aisha K. (2019). Pioglitazone attenuates lipopolysaccharide-induced oxidative stress, dopaminergic neuronal loss and neurobehavioral impairment by activating Nrf2/ARE/HO-1. Neurochem. Res. 44, 2856–2868. doi: 10.1007/s11064-019-02907-0, PMID: 31713708

[ref79] Zhang K. Tarazi F. I. Davids E. Baldessarini R. J. (2002). Plasticity of dopamine D4 receptors in rat forebrain: temporal association with motor hyperactivity following neonatal 6-hydroxydopamine lesioning. Neuropsychopharmacology 26, 625–633. doi: 10.1016/S0893-133X(01)00404-3, PMID: 11927187

[ref80] Zhao Y. Lutzen U. Gohlke P. Jiang P. Herdegen T. Culman J. (2021). Neuroprotective and antioxidative effects of pioglitazone in brain tissue adjacent to the ischemic core are mediated by PI3K/Akt and Nrf2/ARE pathways. J. Mol. Med. 99, 1073–1083. doi: 10.1007/s00109-021-02065-3, PMID: 33864097 PMC8313471

